# Optical Properties of Graphene Nanoplatelets on Amorphous Germanium Substrates

**DOI:** 10.3390/molecules29174089

**Published:** 2024-08-29

**Authors:** Grazia Giuseppina Politano

**Affiliations:** Department of Environmental Engineering, University of Calabria, 87036 Rende, CS, Italy; grazia.politano@unical.it

**Keywords:** thin films, graphene, germanium, ellipsometry, optical properties

## Abstract

In this work, the integration of graphene nanoplatelets (GNPs) with amorphous germanium (Ge) substrates is explored. The optical properties were characterized using Variable-Angle Spectroscopic Ellipsometry (VASE). The findings of this study reveal a strong interaction between GNPs and amorphous germanium, indicated by a significant optical absorption. This interaction suggests a change in the electronic structure of the GNPs, implying that amorphous germanium could enhance their effectiveness in devices such as optical sensors, photodetectors, and solar cells. Herein, the use of amorphous germanium as a substrate for GNPs, which notably increases their refractive index and extinction coefficient, is introduced for the first time. By exploring this unique material combination, this study provides new insights into the interaction between GNPs and amorphous substrates, paving the way for the develop of high-performance, scalable optoelectronic devices with enhanced efficiency.

## 1. Introduction

The scientific community has long been captivated by graphene [1,2], a two-dimensional material known for its extraordinary properties [3,4]. Notably, graphene’s exceptional transparency, conductivity, and flexibility [5] have opened new avenues in material science, suggesting a vast potential yet to be fully explored [6].

This material is characterized by several outstanding intrinsic qualities, such as extraordinary carrier mobility, impermeability to atoms, comprehensive optical absorption, and notable flexibility [7]. These properties position graphene as a promising candidate to drive the evolution of microelectronics in the coming years [8].

The properties of graphene, such as mobility and optical characteristics, can be significantly influenced by the substrate on which it is placed [9]. Moreover, it can be noted that graphene can be used not only in sensors but also as a filter with nanopores, contributing to environmental improvements. Nanopores can be made, for example, as demonstrated in Ref. [10], where few-layer graphene films were nanostructured with swift heavy ions, tuning their electronic and transport properties for potential filtration applications. Similarly, the effects of swift ion tracks on suspended graphene were visualized in Ref. [11], further highlighting the substrate’s role in modifying graphene’s properties.

Recent research focuses on achieving compatibility between graphene and existing silicon-based complementary metal-oxide semiconductor (CMOS) technology [12]. Significant advancements have been made in this direction, particularly with the integration of two-dimensional materials like graphene with three-dimensional semiconductor materials [13]. This combination sis crucial as it improves the interaction between these materials and ensures they work well with existing CMOS technology [14]. CMOS technology primarily uses semiconductors like silicon, germanium, and gallium arsenide, all known for their specific electronic properties at the Fermi level [15]. The successful integration of graphene with these semiconductors highlights the importance of selecting compatible materials in advancing technology, particularly in the semiconductor industry [16].

One notable development in this field is the direct growth of graphene on germanium substrates [17]. This innovation is particularly significant for the semiconductor industry, as it combines graphene’s unique properties with the enhanced mobility of charge carriers in germanium, offering a superior alternative to silicon [18,19]. Research in this area has demonstrated that the interface structure between graphene and germanium is crucial for optimizing optoelectronic applications [16]. The exploration of hybrid composites, particularly those combining germanium (Ge) with carbon-based materials like graphene, has addressed several challenges associated with Ge-based anodes [20]. Moreover, the combination of graphene and germanium shows a marked enhancement in charge capacity, stability, and rate capability, which are essential for lithium-ion battery anodes [21].

In terms of production, methods such as micromechanical cleavage of graphite [22] and chemical vapor deposition [23] have been used in graphene synthesis. However, these techniques have limitations in scaling up for industrial applications. In contrast, graphene-based materials [24], including reduced graphene oxide [25], few-layer graphene [26], multilayer graphene [27], and graphene nanoplatelets (GNPs) [28], offer viable alternatives. GNPs [29], in particular, retain several advantageous properties of single-layer graphene and are produced through economically viable processes, making them suitable for widespread applications.

GNPs [30] present an optimal balance in terms of excellent physical characteristics, scalability in mass production, and cost-effectiveness. The production of GNPs [31] is feasible through various scalable industrial techniques, including wet-jet milling [32], microwave irradiation [33], and liquid exfoliation [34]. Recent research on GNPs has significantly advanced our understanding of their multifunctional applications in many fields. For instance, Wu et al. [35] demonstrated how surface-etched GNPs can reinforce magnesium alloys, leading to improved strength and ductility. The integration of GNPs in sustainable solar desalination systems, as explored by Khoei et al. [36] and Lim et al. [37], highlights their utility in enhancing interfacial evaporation processes for efficient water purification. GNPs have been used in developing advanced composites and wearable sensors, as shown in studies by Dong et al. [38] and Zhu et al. [39], where they contribute to improved mechanical properties and superior thermal management. Additionally, noncovalent functionalization of GNPs has opened new avenues for their application in energy storage devices, particularly in supercapacitors, as reported by Haridas et al. [40].

Herein, GNPs on magnetron-sputtered amorphous germanium thin films were studied using Variable-Angle Spectroscopic Ellipsometry (VASE) [41], a technique that enables precise measurement of the optical constants of these materials, specifically the refractive index and thickness.

The optical model reveals a significant alteration in the optical properties resulting from the interaction between GNPs and the amorphous Ge substrate. The resulting composite exhibits an improved refractive index and extinction coefficient, suggesting a stronger light-matter interaction.

The results presented here are a starting point for the comprehension of interactions between GNPs and amorphous germanium, which could facilitate advancements in nanotechnology and materials engineering.

## 2. Results and Discussion

The optical properties of amorphous germanium substrates were accurately determined by applying the Tauc–Lorentz oscillator [42] using the model implemented in the WVASE32 software. The model applied in this study differs significantly from the Bruggeman approach (see, for example, Ref. [43]). Complete details of the fitting parameters for the substrates are included in the Supplementary Materials.

**Figure 1:**
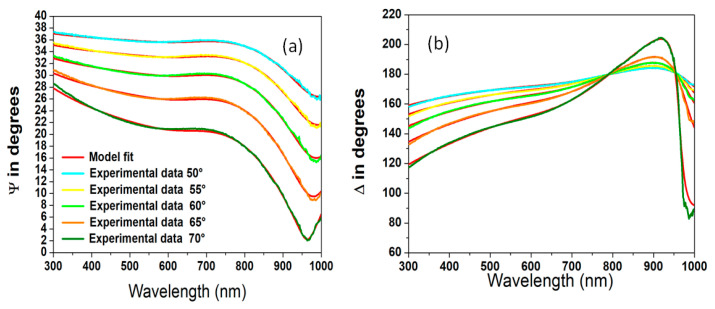
Comparison of experimental and model ψ (**a**) and Δ (**b**) values for germanium substrates at various incidence angles.

Figure 1a,b present both the simulated and measured results for the ψ and Δ spectra across various incident angles within the wavelength range of 300 to 1000 nm for amorphous germanium films.

**Figure 2:**
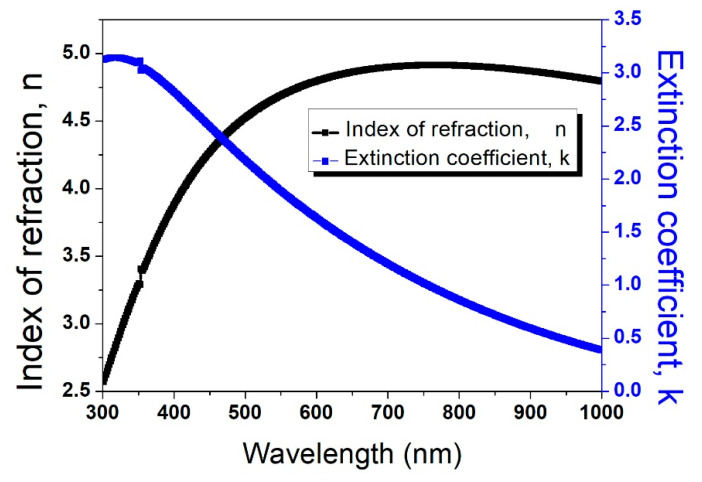
Estimated dispersion laws of germanium substrates. The black curve shows the index of refraction (n), and the blue curve depicts the extinction coefficient (k).

Figure 2 presents the estimated dispersion laws of the optical constants for amorphous germanium thin films. The thickness of the films was estimated to be approximately 100 nm.

**Figure 3:**
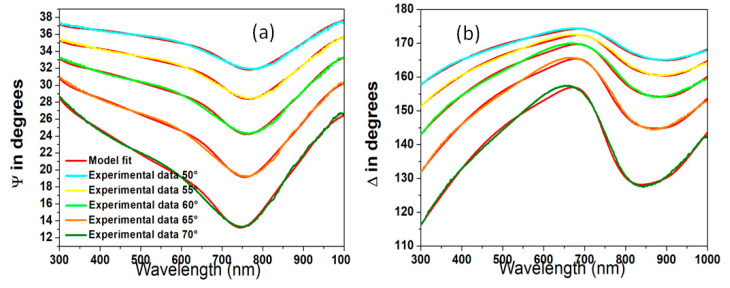
Comparison of experimental and model ψ (**a**) and Δ (**b**) values for GNP films on germanium substrates at various incidence angles.

Figure 3a,b display the measured and calculated values of ψ and Δ for GNP thin films on germanium substrates, covering the wavelength range from 300 to 1000 nm.

A comprehensive fit was conducted across the entire wavelength spectrum. This involved the use of a generalized oscillator model [44], incorporating three Gaussian oscillators to represent the imaginary component of the dielectric constant as detailed in the same source. These oscillators are characterized by three fitting parameters: amplitude, energy position, and broadening. The real part of the dielectric constant was derived using the Kramers–Kronig (KK) [45] relations.

The Gaussian oscillators are described by the following formula:

(1) $$\varepsilon_{2,\mathrm{Gauss}}=A\left[{\exp\left(-\frac{2\sqrt{\ln2}\left(E_{\mathrm{ph}}-E_{c}\right)}{B}\right)}^{2}-{\exp\left(-\frac{2\sqrt{\ln2}\left(E_{\mathrm{ph}}+E_{c}\right)}{B}\right)}^{2}\right]$$

In this formula, *A* represents the amplitude in arbitrary units, *B* denotes the broadening in electronvolts (eV), *E*_ph_ is the photon energy, and *E_c_* signifies the energy position of the oscillator, also in eV [44].

**Figure 4:**
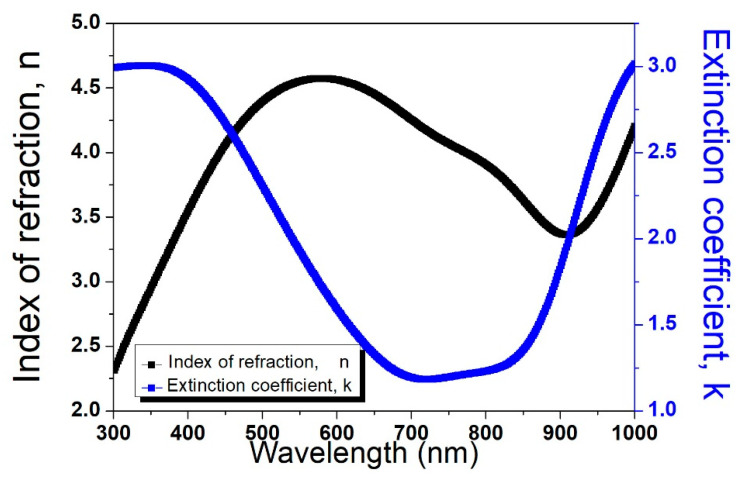
Estimated dispersion laws for GNPs on germanium substrates. The black curve shows the index of refraction (n), and the blue curve depicts the extinction coefficient (k).

Figure 4 shows the estimated dispersion laws for GNPs dip-coated on amorphous germanium substrates.

The thickness of the GNP thin films was determined to be approximately 55 nm.

The MSE obtained was near 6. Table S2 in the Supplementary Materials lists the parameters derived from the optimal fit for GNPs on silicon (reported in Ref. [29]) and for amorphous germanium (present work).

**Figure 5:**
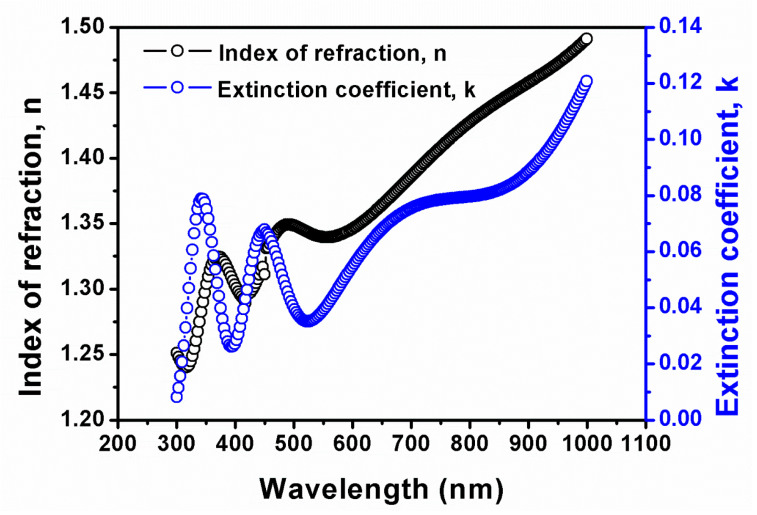
Estimated dispersion laws for GNPs on silicon substrates [29]. The black curve shows the index of refraction (n), and the blue curve depicts the extinction coefficient (k). This content has been reproduced from reference Ref. [29] with permission granted by IOP. Copyright 2019, IOP.

Figure 5 shows the estimated optical properties of GNPs on silicon as published in the author’s previous work [29] for comparative purposes.

As reported in the previous work about GNPs on silicon [29], the oscillator located at 3.7 eV aligns with the surface and interlayer states in graphite’s bulk. The oscillator at 2.7 eV is attributed to defect states, and the one around 1.5 eV corresponds with the predicted π* band in graphite [46].

The amplitude values for the oscillators on amorphous germanium are markedly higher than those on silicon, indicating stronger interactions with the germanium substrate. The broadening (B) and energy position (E) parameters also show variations, indicating differences in the electronic band structure influenced by the substrate. There is also a significant variance in film thickness and the high-frequency dielectric constant between the substrates, emphasizing the influence of amorphous material on the GNP films’ properties.

These differences in parameters underscore the significant impact that the substrate material has on the optical properties of graphene nanoplatelets, influencing their potential applications in various optoelectronic devices.

The enhanced refractive index and extinction coefficient of GNPs on germanium (Figure 4) compared to GNPs on silicon (Figure 5) indicate a stronger light–matter interaction, which may be useful in many optoelectronic applications. In particular, the unexpected jump in the extinction coefficient at approximately 350 nm (Figure 4) can be attributed to electronic transitions within the GNPs. This phenomenon occurs when the energy of the incident photons matches the energy difference between electronic states, leading to increased absorption. The interaction between the GNPs and the amorphous germanium substrate might introduce localized states or modify the density of states at the interface, further contributing to this effect.

E. Aktürk et al. [47] provide an in-depth analysis of how germanium atoms interact with graphene, showing that these atoms preferentially bind at the bridge sites of graphene with substantial binding energy. This interaction induces notable changes in graphene’s electronic structure, shifting it from semimetallic to metallic and generating a magnetic moment. Such modifications are indicative of a strong interaction between the germanium atoms and the graphene lattice, altering its electronic properties.

Applying these insights to the interaction between GNPs and amorphous germanium, it might be that similar strong binding energies and alterations in electronic structure likely occur at the interface between GNPs and the amorphous germanium substrate. The significant optical absorption observed in GNPs on amorphous germanium can be attributed to these electronic structure modifications, which are critical for enhancing the optoelectronic properties of the composite material.

The coupling between the electronic bands of amorphous germanium and GNPs may also lead to strong absorption effects. This coupling likely results from the interaction of electronic states between germanium and graphene, possibly leading to an increased density of states at the Fermi energy and, consequently, enhanced optical absorption.

Moreover, the non-crystalline nature of amorphous germanium might introduce additional complexity into the interaction. For example, the amorphous nature of the germanium differs from a crystalline structure in its ability to introduce localized energy states or disorders that impact electronic interactions. When GNPs are interfaced with amorphous Ge substrates, the peculiarities of amorphous Ge [48], such as its density variations and optical properties, play a significant role in determining the overall behavior of the composite material. Variations in the germanium substrate’s density, porosity, or defect structure may significantly alter the composite material’s optical properties.

High optical absorption of GNPs on amorphous germanium substrates makes them particularly suitable for applications such as optical sensors, photodetectors, and solar cells. In these devices, enhanced optical absorption is desirable for improved efficiency and sensitivity.

## 3. Materials and Methods

The glass substrates underwent a cleaning process using piranha solution, a potent cleaning mixture composed of sulfuric acid (H_2_SO_4_) and hydrogen peroxide (H_2_O_2_).

Germanium films with a thickness of 100 nm were deposited onto glass substrates using a DC magnetron sputtering technique [49] (Edwards Auto306 system, West Sussex, UK) at a working pressure of 4.2 × 10^−2^ mbar, a sputtering power of 40 W, and a sputtering duration of 5 min.

Using X-ray diffraction, no crystalline peaks were observed in the materials, confirming that the Ge layer is amorphous.

The deposition of GNPs onto the prepared germanium films was achieved through a dip-coating process [50]. This procedure was facilitated by a custom-built apparatus, operating at a speed of 0.33 mm/s. The GNPs, with a concentration of 1 mg/mL in water, were purchased from Sigma Aldrich (St. Louis, MO, USA). The composition of the dispersion was 0.1 weight percent graphene and 99.9 weight percent water. The synthesis of the GNPs was achieved through various exfoliation techniques.

In the author’s previous work [29], a detailed analysis of the size distribution of the GNPs’ major and minor lateral dimensions was provided using scanning transmission electron microscopy (STEM).

The analysis yielded average major and minor lateral sizes of approximately 0.05 µm and 0.02 µm, respectively [29].

Variable-Angle Spectroscopic Ellipsometry (VASE) was used to estimate both the thickness and the optical properties *n* (refractive index) and *k* (extinction coefficient) of the GNPs films on germanium samples. Ellipsometric measurements provided precise thickness values and detailed information about the optical properties of the material, enabling a full characterization. The WVASE31 program was used to analyze the ellipsometric data. It employs regression analysis and the Mean Squared Error (MSE) method to fit the model to the experimental data and uses the covariance matrix to provide error bars for the measured values. The ellipsometric parameters, ψ and Δ, were measured using a J.A. M2000 F (Woollam Co., Lincoln, NE, USA) rotating compensator ellipsometer. This measurement spanned a wavelength range of 300–1000 nm, at incident angles varying from 50° to 70° in 5° increments, all conducted at room temperature. The optical model and optimal parameter values for the films were determined using the WVASE32 [44] software from J.A. Woollam, which focuses on minimizing the MSE.

## 4. Conclusions and Outlook

In this study, GNP films were dip-coated onto magnetron-sputtered amorphous germanium substrates. The optical properties and the thickness of the films were studied using VASE. One of the crucial findings from the ellipsometric data analysis is the observation of a higher refractive index and extinction coefficient of the GNPs on amorphous germanium compared to GNPs on silicon substrates. This indicates a significant change in the optical properties due to the interaction between GNPs and the amorphous germanium substrate. The enhanced refractive index and extinction coefficient are indicative of a stronger light–matter interaction, which may be useful in many optoelectronic applications. Such interaction, which likely involves the merging of electronic states from both materials, might cause a rise in the density of states at the Fermi level, thereby boosting the optical absorption.

These results could be useful for future studies aimed at exploring the full potential of GNP–amorphous germanium composites. The increased absorption and improved light interaction make GNPs on amorphous germanium a suitable candidate for applications requiring high optical sensitivity and efficiency. The potential applications of this graphene–germanium composite are promising for advanced photodetectors, high-efficiency solar cells, and innovative optical sensors.

However, the scalability of the dip-coating process for GNPs and the magnetron sputtering technique for amorphous germanium deposition in industrial applications remains a challenge. Additionally, the long-term stability and durability of the GNP–germanium interface under several operational conditions are aspects that require further investigation.

## Data Availability

Data are contained within the article and Supplementary Materials.

## Supplementary Materials

The following supporting information can be downloaded at: https://www.mdpi.com/article/10.3390/molecules29174089/s1, Figure S1: Scanning transmission electron microscopy image displaying graphene nanoplatelets drop-cast on a gold mesh. Figure S2: Image highlighting the specific marked areas of the graphene nanoplatelets on the gold mesh. Figure S3: Distribution of size (depicted as a histogram and fitted with an exponential function) for the minor (a) and major (b) lateral dimensions of graphene nanoplatelets. Table S1: Tauc-Lorentz oscillators parameters obtained from the best fit of ellipsometric experimental data for germanium/glass substrates. *D*, *A*, *E_0_*, *C*, and *E_g_* are the film thickness, amplitude, peak position, broadening, and optical band gap. Table S2: Parameters of Gaussian oscillators derived from the most accurate fit for graphene nanoplateletes thin films on amorphous germanium and on silicon substrates [24]: amplitude (A), broadening (B), energy position (E), film thickness (d) and high-frequency dielectric constant *ε*_∞_.
