# Supplementary material for: Optical Properties of Graphene Nanoplatelets on Amorphous Germanium Substrates
Source: Molecules. 2024 Aug 29;29(17):4089. doi: 10.3390/molecules29174089 (PMC11397050; doi:10.3390/molecules29174089)
Supplement: Supplementary file 1 [file molecules-29-04089-s001.zip › molecules-3177293-supplementary.pdf]

# Supplementary materials

## 1. STEM images

Figure S1 presents a Scanning transmission electron microscopy (STEM) image of GNPs that have been deposited onto a gold mesh. Figure S2 displays the result of applying a threshold algorithm using ImageJ software to the original STEM image.

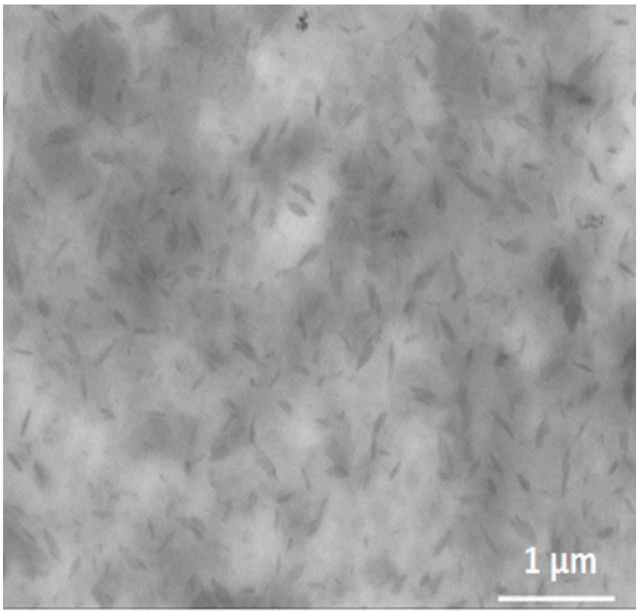


Figure S1: Scanning transmission electron microscopy image displaying graphene nanoplatelets drop-cast on a gold mesh.

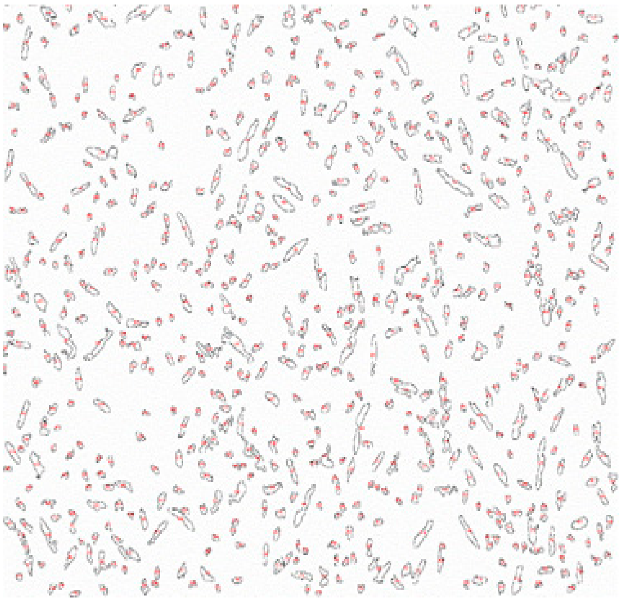

Figure S2: Image highlighting the specific marked areas of the graphene nanoplatelets on the gold mesh.

Figures S3(a) and S3(b) display the size distributions for the minor and major lateral dimensions of GNPs, which are represented as ellipses.

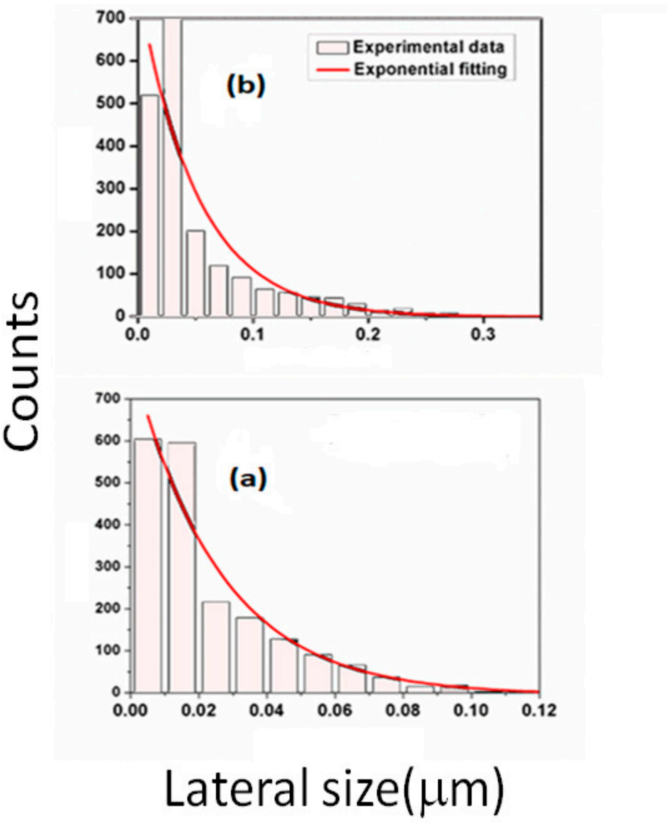


Figure S3: Distribution of size (depicted as a histogram and fitted with an exponential function) for the minor (a) and major (b) lateral dimensions of graphene nanoplatelets.

The analysis yielded average major and minor lateral sizes of approximately 0.05 µm and 0.02 µm, respectively.

## 2 VASE models

In a preliminary study, the optical models of the substrates (magnetron sputtered germanium films/glass) were estimated. The float glass substrates were described by an optical

Cauchy model.

The dielectric response of germanium films on glass substrates was described with Tauc-Lorentz (TL) dispersion model. The imaginary part of the TL dielectric function is expressed as:

$$\varepsilon_{2TL}(E) = \frac{AE_0 C(E - E_G)^2}{(E^2 - E_0^2)^2 + C^2 E^2} \frac{1}{E} \qquad E > E_g$$

$$\varepsilon_{2TL}(E) = 0 \qquad E \leq E_g \qquad \text{(S1)}$$

The four fitting parameters $A$, $E_0$, $C$, and $E_g$ are the amplitude, peak position, broadening, and optical band gap and all are in units of energy. The real part of the dielectric function is then obtained through Kramers-Kronig integration.

Table S1 shows a scheme of the best fit parameters for germanium/ glass substrates.

**Table S1:** Tauc-Lorentz oscillators parameters obtained from the best fit of ellipsometric experimental data for germanium/glass substrates. *D, A,* $E_0$*, C, and* $E_g$ are the film thickness, amplitude, peak position, broadening, and optical band gap.

| D(NM) | $(eV)$ | $E_0$ $(eV)$ | C $(eV)$ | $E_G$ $(eV)$ |
|---|---|---|---|---|
| 100±3 | 272.0±0.7 | 2.6±0.1 | 8.6±0.1 | 0.950±0.001 |

Table S2 lists the parameters derived from the optimal fit for GNPs on silicon (reported in Ref. [24]) and for amorphous germanium ( present work). It is important to note that the ellipsometric model of GNPs on silicon does not depend on the thickness of the films (Tompkins, H.; Irene, E.A. Handbook of Ellipsometry; William Andrew: Norwich, NY, USA, 2005).

**Table S2:** Parameters of Gaussian oscillators derived from the most accurate fit for graphene nanoplateletes thin films on amorphous germanium and on silicon substrates [24]: amplitude (A), broadening (B), energy position (E), film thickness (d) and high-frequency dielectric constant $\varepsilon_\infty$.

| | **GERMANIUM** | **SILICON**([24]) |
|---|---|---|
| $d$ (nm) | 55.0±0.1 | 8.0±0.1 |
| $\varepsilon_\infty$ | 2.5±0.1 | 3.7 ±0.2 |

| | | |
|---|---|---|
| $A_1$ | 158.4±1.6 | 0.20±0.01 |
| $B_1(eV)$ | 2.60±0.02 | 0.60 ±0. 02 |
| $E_{c1}(eV)$ | 3.5±0.1 | 3.7 ±0. 1 |
| $A_2$ | 17.6±0.9 | 2.1±0.1 |
| $B_{c2}(eV)$ | 0.91±0.02 | 0.40±0. 01 |
| $E_2(eV)$ | 2.5±0.1 | 2.7 ±0. 1 |
| $A_3$ | 7.10±0.07 | 0.20±0.01 |
| $B_{c3}(eV)$ | 0.19±0.01 | 1.2 ±0.1 |
| $E_{c3}(eV)$ | 1.2±0.1 | 1.5 ±0. 1 |
